# The Role of the Craniocervical Junction in Craniospinal Hydrodynamics and Neurodegenerative Conditions

**DOI:** 10.1155/2015/794829

**Published:** 2015-11-30

**Authors:** Michael F. Flanagan

**Affiliations:** ^1^American Chiropractic Association, 1701 Clarendon Boulevard, Suite 200, Arlington, VA 22209, USA; ^2^American Chiropractic Neurology Board, 3710 Robinhood Drive, Temple, TX 76502, USA

## Abstract

The craniocervical junction (CCJ) is a potential choke point for craniospinal hydrodynamics and may play a causative or contributory role in the pathogenesis and progression of neurodegenerative diseases such as Alzheimer's disease, Parkinson's disease, MS, and ALS, as well as many other neurological conditions including hydrocephalus, idiopathic intracranial hypertension, migraines, seizures, silent-strokes, affective disorders, schizophrenia, and psychosis. The purpose of this paper is to provide an overview of the critical role of the CCJ in craniospinal hydrodynamics and to stimulate further research that may lead to new approaches for the prevention and treatment of the above neurodegenerative and neurological conditions.

## 1. Introduction

The CCJ comprises the base of the skull, atlas (C1), and axis (C2), as well as muscles and connective tissues that connect the skull to the cervical spine. It further includes the dura mater and dentate ligament attachments of the brain and cord to the foramen magnum and upper cervical spine [1–8]. Moreover, the CCJ links the vascular and cerebrospinal fluid (CSF) systems in the cranial vault to those in the spinal canal. Craniospinal hydrodynamics refer to the relationship between blood and CSF volume, pressure, and flow in the relatively closed confines of the compartments of the cranial vault and spinal canal. Malformations and misalignments of the CCJ cause deformation and obstruction of blood and CSF pathways and flow between the cranial vault and spinal canal that can result in faulty craniospinal hydrodynamics and subsequent neurological and neurodegenerative disorders.

While the exact source, such as the arteries at the base of the brain or choroidal vessels, is still debated, in either case, it is generally accepted that craniospinal hydrodynamics are primarily driven by arterial pulsations [9–19] and further modified by respiration, Valsalva maneuvers, body movements, upright posture, inversion, and recumbent position. The arterial pulsations pump a relatively large volume of blood into the brain and cord during systole causing a spike in intracranial pressure (ICP). To prevent damage to the brain from excess pulsatility pressure, the increase in volume must be matched by a simultaneous increase in drainage of venous blood and CSF from the cranial vault. In addition to the internal jugular veins humans developed an accessory drainage system comprising emissary veins that link the dural sinuses to the vertebral venous plexus and serve as the primary drainage routes of the brain during upright posture [20–30]. Because the dural sinuses, facial veins, and vertebral veins have no valves, the direction of venous blood flow is determined by hydrostatic pressure gradients. The pressure gradients produced by upright posture cause blood to flow preferentially from the dural sinuses into the vertebral veins. Conversely, inversion reverses the pressure gradient causing blood to flow from the vertebral veins to the dural sinuses, which increases intracranial and intraocular pressures [20–30]. CSF flow between the subarachnoid space in the cranial vault and spinal canal is likewise determined by the same pressure gradients.

There are many different types of malformations and anomalies of the CCJ such as atlantooccipital assimilation, basiocciput hypoplasia, malformations of the condyles, and malformations of the dens [31]. Certain types of malformations and anomalies of the CCJ, such as Chiari malformations [32–36], abnormal clivoaxial angle [37–41], hypoplasia of the posterior fossa [42–47], basilar invagination, and platybasia [48–51] are predisposed to coning of the cerebellar tonsils into the foramen magnum that can block blood and CSF flow between the cranial vault and spinal canal resulting in hydrocephalus. Other malformations, such as hypoplasia of the foramen magnum, hypoplasia of the jugular foramen, and anomalies of the odontoid process of axis can also affect craniospinal hydrodynamics and cause hydrocephalus [37–41, 52–56]. Premature closure of the sutures of the cranial vault, called craniosynostosis, especially craniosynostosis of the lambdoid suture, has also been associated with hypoplasia and crowding of the posterior fossa, Chiari malformations, and hydrocephalus [54–60]. Craniofacial anomalies such Crouzon, Pfeiffer, Apert, and Saethre-Chotzen are likewise often associated with malformations of the base of the skull and Chiari malformations that can block CSF flow resulting in hydrocephalus [61–65]. Connective tissue disorders, such as rheumatoid arthritis and Ehlers-Danlos syndrome, can cause tears, degeneration, ligament laxity, and cranial settling. Cranial settling causes the skull to rock back on the CCJ and sink onto the upper cervical spine during upright posture [66, 67]. In contrast to the brainstem getting pushed down into the foramen magnum as occurs in Chiari malformation, cranial settling shifts the position of the CCJ upward relative to the brainstem. The outcome is similar to basilar invagination and can cause hydrocephalus.

In addition to blocking CSF flow and causing hydrocephalus, malformations, anomalies, and misalignments of the CCJ can obstruct blood flow through the vertebral arteries [59–63] and veins that can lead to chronic ischemia and edema. Furthermore, chronic edema decreases perfusion pressure and arterial flow in the brain which can result in oxidative stress and chronic ischemia as well. Oxidative stress and chronic ischemia have been implicated in the cause of atrophy of the brain. Many authors have also maintained that venous insufficiency can cause hydrocephalus [68–79]. Moreover, venous insufficiency and hydrocephalus can lead to increased pulsatility, which is a major suspect in hydrocephalus and ventriculomegaly [80–84]. It is possible, therefore, that blockage of blood and CSF flow due to malformations and misalignments of the CCJ may play a role in chronic ischemia, edema, hydrocephalus, and ventriculomegaly.

In brief, the CCJ is a potential choke point for blood and CSF flow between the cranial vault and spinal canal that can cause faulty craniospinal hydrodynamics and subsequent chronic ischemia, edema, and hydrocephalus. Upright posture and motion of the cervical spine compound the strains and deformation of blood and CSF pathways of the CCJ further contributing to blockage of flow. Faulty craniospinal hydrodynamics have been associated with Chiari malformations [31–36], craniosynostosis [54–60], craniofacial anomalies [61–65], anomalies of the CCJ [31–53, 85–87], and Dandy-Walker complex in children [88–90]. Faulty craniospinal hydrodynamics may also play a role in neurodegenerative diseases such as Alzheimer [91–97], Parkinson [98–101], multiple sclerosis [102–108], dementia [109–113], and motor neuron diseases [114–121]. Lastly, faulty craniospinal hydrodynamics may play a role in migraines [122–128], silent-strokes [128–130], seizures [131–134], psychosis [135–145], depression [146–148], and mania [149–153]. The aim of this paper is to stimulate further research into the role of the craniocervical junction and faulty craniospinal hydrodynamics in neurodegenerative and other neurological conditions that will lead to prevention and more effective treatments.

## 2. Arteries and Veins of the CCJ

The arteries and veins of the CCJ play key roles in craniospinal hydrodynamics. The main arteries that pass through the CCJ include the vertebral and spinal arteries. The venous system of the CCJ includes the (1) dural sinuses of the posterior fossa, (2) suboccipital cavernous sinus, (3) occipital-marginal sinuses, (4) emissary veins, and (5) vertebral veins.

The vertebral arteries make up the posterior blood supply routes that provide about twenty percent of blood flow to the brain. The twenty percent of blood flow they supply includes most of the diencephalon (thalamus and hypothalamus) and all of the brainstem, as well as the medial temporal and occipital lobes. The internal carotid arteries are the anterior supply routes and supply about eighty percent of blood flow to the brain including most of the lobes and some of the diencephalon.

The circle of Willis encircles the infundibulum located above the sella turcica and links the anterior blood supply routes to the posterior blood supply routes. There are variations in the layout of the circle. In some cases it is incomplete. The design of the circle of Willis affects its ability to supply collateral circulation to the brain. It also affects the direction of arterial flows in the anterior and posterior supply routes under normal and pathological conditions, such as steal syndromes involving the vertebral arteries.

**Figure 1:**
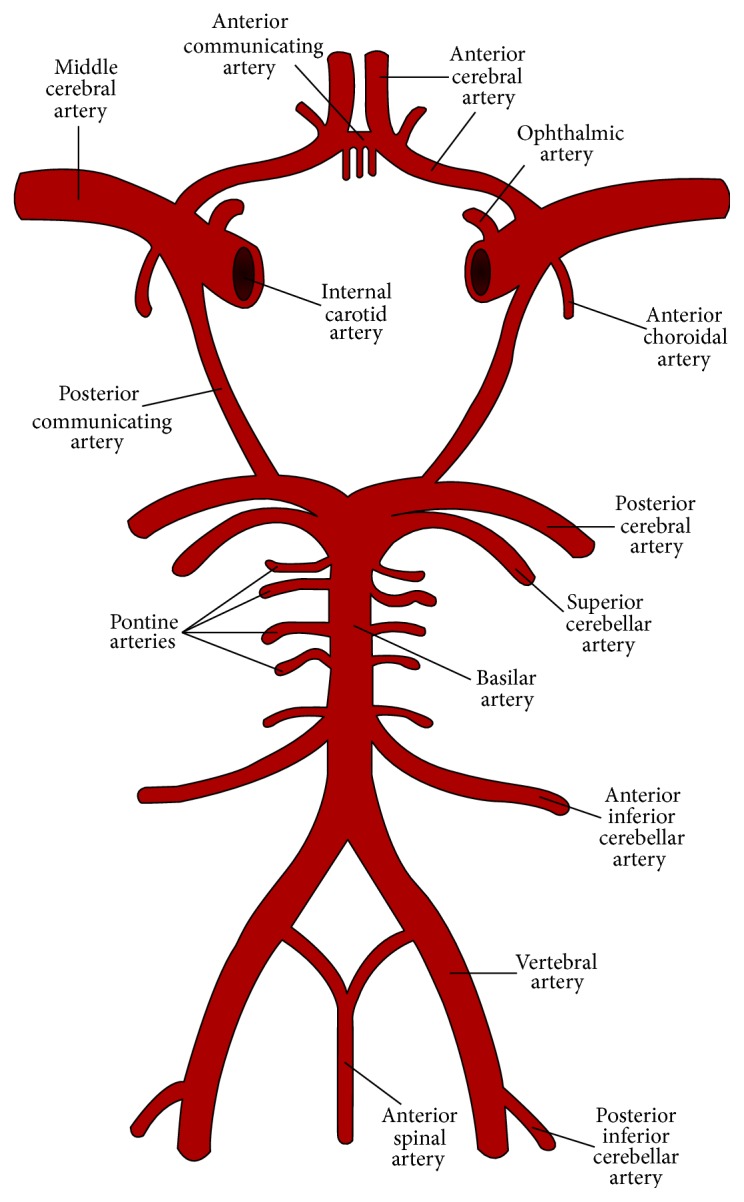
Vertebral-basilar arteries and circle of Willis.

The vertebral arteries arise from the proximal segments of the subclavian arteries and are broken down into four segments. The first segment is from the origin at the subclavian artery to where it enters the intervertebral foramen of C6 or C5. The second segment passes through the intervertebral foramen from C6 up to C2. The third segment of the vertebral artery passes through the intervertebral foramen of the CCJ, turns back around the condyles, and then pierces the suboccipital cavernous sinus of the posterior atlantooccipital membrane through which it travels toward the midline of the occiput. As they approach the midline of the occiput the vertebral arteries turn cephalward and pass through the foramen magnum. After passing through the foramen magnum the vertebral arteries penetrate the dura mater and enter the brain. The fourth segment continues from where the vertebral arteries enter the brain up to the pontomedullary junction where they unite to become the basilar artery (see Figure 1).

Subclavian steal syndromes occur when the proximal portion of the subclavian artery is obstructed due to stenosis resulting in decreased blood flow and pressure downstream in the vertebral and axillary arteries. This creates a pressure gradient that causes the arm to draw blood from the ipsilateral vertebral artery, which in turn gets its supply from the contralateral vertebral artery via their connection to the basilar artery [153–161]. Subclavian steal syndromes can in turn steal blood flow from upstream vessels of the brain including the ophthalmic arteries of the eyes.

The ophthalmic arteries are the first branches of the internal carotid artery. Decreased pressure in the circle of Willis relative to arterial pressure in the ophthalmic artery can draw blood away from the eye to the ipsilateral brain. Consequently, relatively lower downstream pressure in the posterior portion of the circle of Willis, basilar, and vertebral arteries, which occurs in a subclavian steal syndrome can result in an ophthalmic steal syndrome and hypoperfusion of the optic nerve head (see Figure 1). Hypoperfusion of the optic nerve head can cause a loss of vision, which occurs in certain cases of multiple sclerosis associated with amaurosis fugax [162–164], as well as in retinal migraine headaches [165–167]. Hypoperfusion of the optic nerve head may also play a role in low tension glaucoma [168–171]. Interestingly, researchers have shown that the design of the eye also affects glaucoma. Patients with smaller globes relative to the size of the optic nerve appear to be more susceptible to glaucoma as a consequence of crowding and congestion of the nerve similar to hypoplasia of the posterior fossa causing crowding and congestion of the brainstem and cerebellum and predisposition to Chiari malformations associated with hydrocephalus.

It is further interesting to note that migraines have been associated with hyperintensity signals on brain scans that indicate ischemic damage. The hyperintensity signals are often found in the distribution of the posterior blood supply, especially the cerebellum [128–130, 172, 173]. The vertebral arteries also supply the medial temporal and occipital lobes. Decreased blood flow to the medial temporal and occipital lobes may play a role in the déjà vu and aura symptoms associated with migraines. It seems plausible that malformations, misalignments, and deformation of the CCJ and dura mater can obstruct blood flow through the vertebral arteries resulting in ischemia and signs and symptoms associated with of migraine headaches.

In addition to providing twenty percent of blood flow to the brain, the vertebral arteries provide a significant portion of the blood supply to the cord via the anterior and posterior spinal arteries. The anterior spinal arteries typically arise from the fourth segment of the vertebral arteries after the posterior inferior cerebellar arteries (see Figure 1). The posterior spinal arteries are the first branches of the anterior spinal arteries. Alternatively, in some cases the posterior spinal arteries arise from the posterior inferior cerebellar arteries. The two anterior spinal arteries unite while the posterior spinal arteries remain paired as they descend through the CCJ to supply the anterior and posterior aspects of the cord. Long and short circumferential arteries branch off from the anterior spinal arteries to supply the dorsolateral surface of the medulla and upper cervical cord. It seems plausible that malformations, misalignments, and deformation of the CCJ can obstruct blood flow through the anterior and posterior spinal arteries resulting in decreased blood flow to the cord.

Recent clinical evidence by Rafael further suggests that ALS may be due to progressive ischemia in the intraparenchymal territory of the anterior-ventral spinal arteries and/or the anterior spinal artery. The anterior-ventral spinal arteries are branches of the inferior thyroid and ascending cervical arteries that stem from the thyrocervical trunk, which arises from the subclavian artery distal to the vertebral artery. The anterior spinal and anterior-ventral spinal arteries supply the ventral aspect of the cord. Rafael found some key common characteristics in ALS patients based on CT and MRI scans of the skull and cervical spine, as well as from observations made during surgery that included (1) anatomical variants of the fourth segment of the vertebral arteries; (2) atherosclerosis of the fourth segment of the vertebral arteries, as well as the basilar artery; (3) atrophy of the pyramids; (4) atrophy of the frontoparietal cortex; (5) questionable zones that appeared to be microinfarcts in the medulla oblongata and cervical cord; (6) exsanguinated short and long circumferential arteries that branch off from the anterior-ventral spinal and/or the anterior spinal arteries; (7) atrophy of small nerve roots in cranial nerves IX, X, and XI; and (8) degeneration of the cervical spine. Based on his clinical and neurosurgical observations, as well as the following omental transplantation for revascularization, Rafael posits that the primary cause in certain cases of olivopontocerebellar atrophy (Parkinson's plus), ALS, and possibly central pontine myelinolysis is ischemic in origin and related to vascular anomalies and atherosclerotic plaques located at the mouths of the collateral branches from the vertebral and basilar arteries [119, 121].

Many authors have also suggested that ALS may be related to trauma, especially head trauma [114–120]. Among other things, trauma can tear ligaments of the craniocervical junction resulting in joint instability and misalignments. Misalignments due to degeneration and ligament laxity are also associated with connective tissue disorders such as rheumatoid arthritis and Ehlers-Danlos [66, 67, 174], which have in turn been associated with cranial settling causing a similar effect to basilar invagination mentioned previously. Trauma superimposed on malformations, anomalies, and degenerative changes in the arteries and spine may further compromise circulation in the distribution of the vertebral-basilar arteries. It is possible that obstruction to blood flow through the anterior and posterior spinal arteries due to malformations and misalignments of the CCJ may play a causative or contributory role in neurodegenerative conditions of the cord such as ALS.

**Figure 2:**
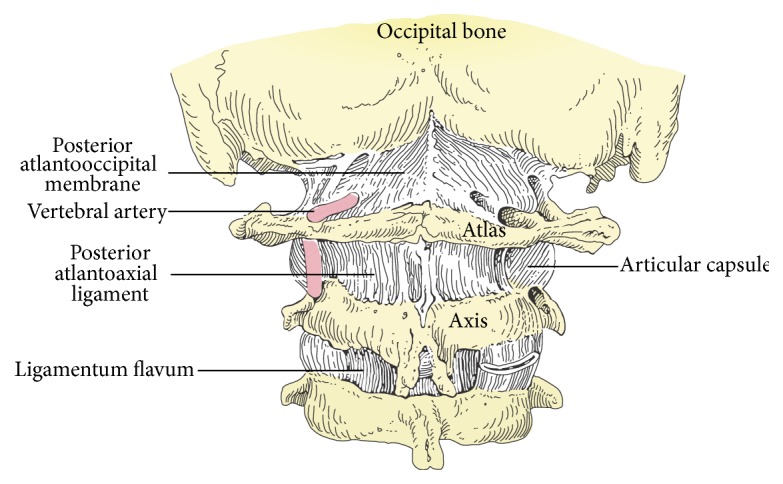
CCJ and suboccipital cavernous sinus.

The third segment of the vertebral artery passes through the suboccipital cavernous sinus (also known as the posterior atlantooccipital membrane) of the CCJ (see Figure 2). The extracranial suboccipital cavernous sinus is the homolog of the intracranial cavernous sinus [175], which contains the internal carotid artery. The cavernous and suboccipital cavernous sinuses are part of the dural sinus system of the brain.

**Figure 3:**
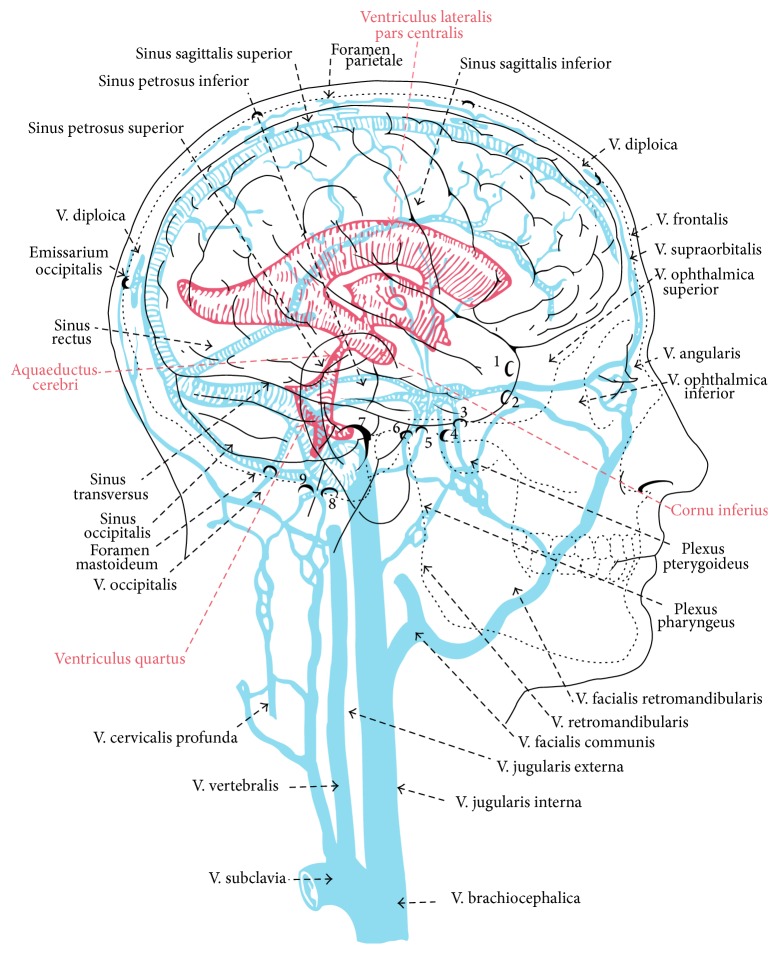
Ventricles, sinuses, and veins.

The dural sinuses are the large drainage veins of the brain (see Figure 3 striped blue lines). They are not true veins. Instead, they are tunnels formed from dura mater lined with endothelium of veins. The outer coat of dura mater makes them stronger than veins and better suited to resist deformation by the brain, as well as collapse due to sudden drops and negative pressures caused by upright posture. The dural sinuses have no valves to check inversion flows and are connected to an extensive system of valveless emissary, scalp, face, and diploic and vertebral veins (see Figure 3 bold semicircles at top and bottom of the skull). The diploic veins are located between the inner and outer cortices of the skull. Emissary veins link the dural sinuses to the diploic and extracranial veins including the valveless veins of the vertebral venous plexus of the spine and spinal canal (see Figure 3 V. vertebralis). The direction of venous blood flow in this vast system of interconnected valveless veins is determined by hydrostatic pressure gradients that are affected by body movements, respiration, Valsalva maneuvers, upright posture, and inversion [22–30]. Although it lies outside the cranial vault, the suboccipital cavernous sinus is structurally similar and has connections to the dural sinuses inside the cranial vault.

The cavernous and suboccipital cavernous sinuses are also part of the cerebral autoregulatory and brain cooling mechanisms. As part of the cerebral autoregulatory system the cavernous and suboccipital cavernous sinuses contain chemo- and baroreceptors used to maintain steady blood flow in the brain by way of vasoconstriction or relaxation [25, 28, 175–180]. Their role in brain cooling is to serve as countercurrent heat exchangers that bath the carotid and vertebral arteries in venous blood that has been cooled at the surface by conduction, convection, radiation, and sweating. The cavernous sinuses are primarily cooled by the eyes and ophthalmic veins. The suboccipital cavernous sinus is cooled by emissary veins from the scalp, face, and neck, as well as diploic veins located between the inner and outer cortices of the cranial vault. The diploic veins are similarly cooled by surface veins and provide an additional layer of thermal protection for the cranial vault. The diploic veins and countercurrent heat exchange system keep the brain two to three degrees cooler than the rest of the body [175, 179, 180].

The occipital-marginal sinuses of the CCJ are part of the dural sinus system of the brain that evolved in primates, hominids, and humans to accommodate increased venous outflow that occurs during upright posture by increasing the capacity and efficiency of the drainage system. The occipital sinus is connected to the torcula, also known as the confluence of sinuses, located adjacent to the internal occipital protuberance. The torcula links the superior and inferior sagittal sinuses to the transverse sinuses. There are several variations of the torcula and occipital sinus connections. In some cases they are absent. The marginal sinus is located around the rim of the foramen magnum. The occipital-marginal sinuses connect to the vertebral venous plexus of the spine and spinal canal through the CCJ. Although it plays a less prominent role, the occipital-marginal sinus system still persists in humans [25].

More than the occipital-marginal sinuses, human evolution tended to favor the development of a robust system of emissary veins to connect to the vertebral venous plexus. Some of the emissary veins pass through emissary foramen in the cranial vault such as the parietal, sphenoid, mastoid, and occipital emissary foramen, as well as others. The most favored and well developed emissary venous outlets used to drain the brain during upright posture in humans, however, pass through the foramen magnum and hypoglossal and condylar canals of the CCJ [22–30]. These outlets are located in the bottom of the posterior fossa below the level of the jugular foramen when the head is held upright. The lower location of the emissary venous outlets in and around the foramen magnum makes them more suitable for draining the bottom of the bowl during upright posture than the internal jugular veins.

**Figure 4:**
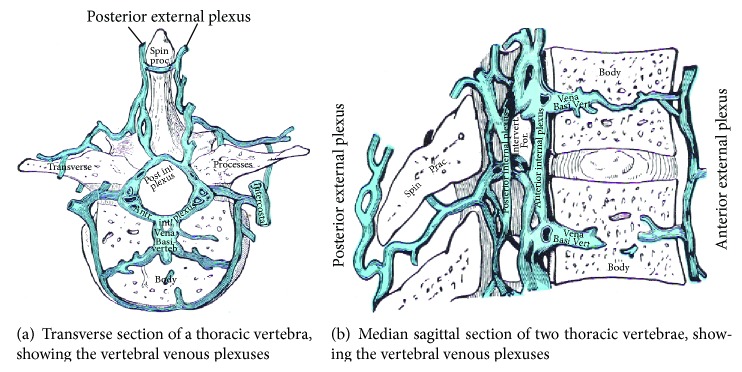
Vertebral venous plexus.

The occipital-marginal sinus, emissary, and hypoglossal and condylar venous routes all connect to the vertebral venous plexus. The vertebral venous plexus is an extensive network of valveless veins located along the length of the spine and inside the spinal canal with connections between them (see Figures 3 and 4 V. vertebralis). The veins of the internal vertebral venous plexus are located in the epidural space between the cord and spinal canal. This makes the internal vertebral venous plexus the homologue of the cortical or bridging veins located between the brain and the cranial vault. Blood flow in the epidural vertebral veins is affected by motion, respiration, Valsalva maneuvers, abdominal pressure, upright posture, and inversion. It is also affected by spondylosis, scoliosis, and stenosis in the lower spine [22–30].

Malformations and misalignments of the CCJ cause strains and deformation of the dura mater and suboccipital cavernous sinus, which can affect circulatory pathways and blood flow in the brain. Spondylosis, stenosis, and scoliosis in the lower spine cause deformation of the epidural space of the spinal canal, as well as deformation of the dura mater (thecal sac) that can similarly affect blood flow in the vertebral veins and cord. In addition to straining the dura of the CCJ, cervical kyphosis (reversal of the normal lordotic curve) also causes the internal jugular vein to bend around the transverse process of atlas, reversing its normal course, as well as potentially compressing the vein and increasing resistance to blood flow. In brief, malformations and misalignments of the CCJ may play a role in chronic ischemia and edema, which may in turn lead to neurodegenerative processes and subsequent diseases.

## 3. The CCJ and CSF Flow

**Figure 5:**
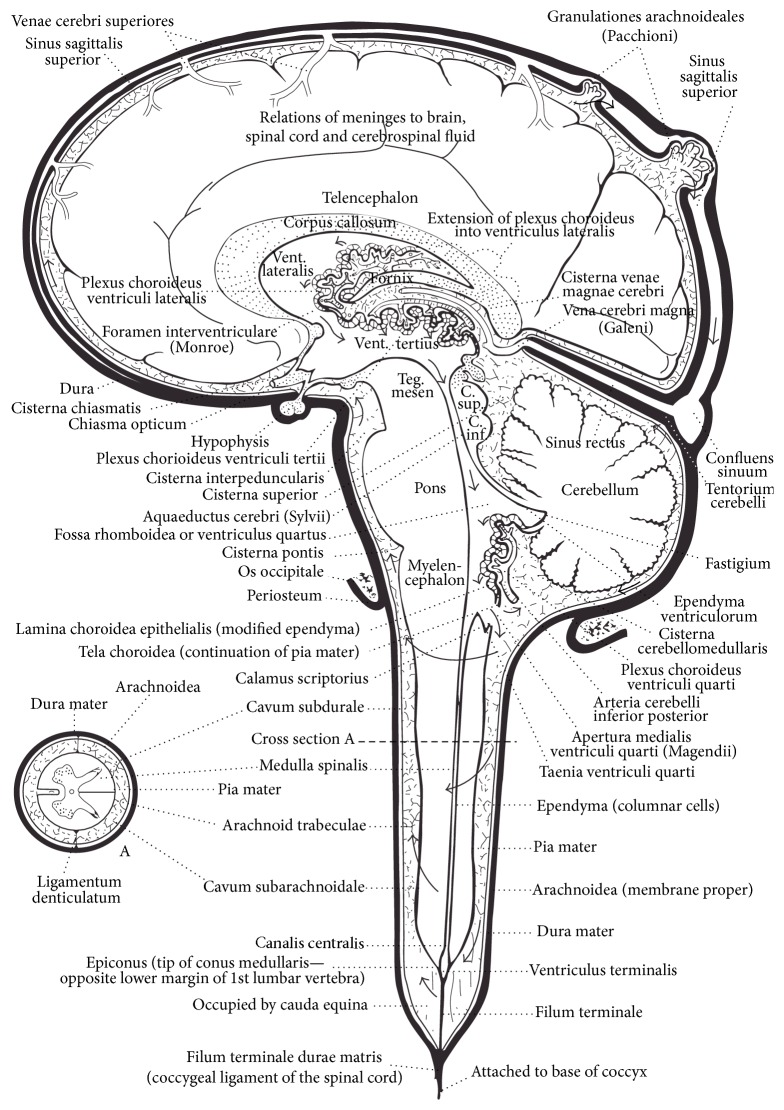
CSF circulation.

CSF is produced in the ventricles. The lateral ventricles are the highest and largest ventricles located in the left and right hemispheres beneath the corpus callosum. CSF flows from the lateral ventricles through the foramen of Monroe into the third ventricle located within the walls of the thalamus of the diencephalon. It flows out of the third ventricle through the cerebral aqueduct of Sylvius in the midbrain and into the fourth ventricle located anterior to the cerebellum and posterior to the pons and medulla. CSF flows out of the fourth ventricle through four openings. The foramen of Magendie, located in the midline of the fourth ventricle, and the two foramens of Luschka, located on the lateral walls, drain into the prepontine cistern and cisterna magna. The central canal of the medulla and cord is connected to the obex of the fourth ventricle. The central canal of the cord is the embryological remnant of the neural tube and homolog of the ventricles and cerebral aqueduct of Sylvius. Although it is often discontiguous in adults, the central canal passes through the center of the lower brainstem and spinal cord and terminates as a cistern in the conus medullaris (see Figure 5).

The cisterns are expansions of the subarachnoid space (see Figure 5, stippled gray area around the brain). The subarachnoid space is located between the arachnoid and pia mater of the leptomeninges and serves as a conduit for CSF flow and for the passage and protection of the large arteries and veins of the brain and cord. The subarachnoid space is fortified with struts called trabecula to maintain its structural integrity and patency to protect the blood vessels within from compression. The cisterns are strategically located to provide extra protection and buoyancy for the brain, especially the brainstem due to its location above the base of the skull and foramen magnum. The cisterna magna is the lowest cistern located beneath the cerebellum above the foramen magnum. The premedullary cistern is located ventral to the medulla. The lower cisterns, such as the cisterna magna, premedullary, and prepontine cisterns, help to prevent the cerebellum from sinking into the foramen magnum, which is called a pressure cone [181–185]. Pressure cones cause cerebellar tonsillar ectopia similar to Chiari malformations.

The correct volume and pressure of CSF in the brain are also important for turgor, which is necessary to maintain openings, pathways, separation, and spatial orientation of the different parts of the brain [186, 187], as well as prevent the brain from sagging and sinking. Proper CSF pressure also plays a role in the development of the ventricles, cerebral aqueduct, foramen, cisterns, and subarachnoid space. In fact, low CSF pressure may be detrimental to the formation and development of the outlets of the fourth ventricle. Delays and maldevelopment of the foramen of the fourth ventricle can cause Dandy-Walker complex [88–90]. More importantly, CSF buffers incoming arterial pulsations and pressure waves. It also prevents the veins in the brain from collapsing due to negative pressure that occurs during upright posture. It does so due to a simultaneous drop in CSF pressure in the subarachnoid space, which offsets the drop in pressure inside the veins.

CSF is produced in the brain by active and passive means. Both active and passive production draw CSF from blood contained in the capillaries of the choroid plexuses of the ventricles. The choroid plexuses are covered by a layer of ependymal columnar salt secreting cells. Although it is far more complex, in brief, active production is a continuous process that secretes salts from the ependymal cells into the ventricles. The salts draw blood out of the choroid plexus by osmosis across the extra-fine filter of the blood brain barrier. CSF is thus an extra-fine filtrate of the blood. More recent research shows that a relatively large portion of CSF also comes from interstitial fluids that flow through the blood brain barrier driven by concentration and pressure gradients, as well as more active means such as through aquaporin channels [188–204].

The passive production of CSF uses hydrostatic pressure gradients. Passive production occurs during upright posture which decreases venous pressure in the vertebral veins and dural sinuses. The decrease in pressure in the superior sagittal sinus increases the CSF pressure gradient. The increase in the CSF pressure gradient draws additional blood from the choroid plexus, which is called passive production. The increase in passive production is necessary to make up for the loss of volume due to increased CSF outflow to prevent coning of the brainstem.

The cisterna magna and premedullary cisterns of the posterior fossa are connected to the subarachnoid space of the cord through the CCJ. The subarachnoid space of the cord and lumbar cistern, which is an enlargement of the subarachnoid space in the lumbar spine, serves as an expansion tank for excess CSF intracranial volume and pressure. Its role as an expansion tank is important in order to maintain proper CSF volume, which is essential to prevent coning of the brainstem. Two common surgical procedures known to cause coning are excess drainage by CSF shunts and spinal taps (lumbar puncture). Trauma can also cause dural tears resulting in CSF leaks and coning. Connective tissue disorders such as Ehlers-Danlos appear to be predisposed to CSF leaks as well. In addition to preventing coning, proper CSF volume is essential to the regulation of intracranial pressure (ICP), which increases exponentially with increases in volume.

Chiari malformations are typically attributed to malformations of the brainstem in children in which the lower portion of the cerebellum, called the tonsils, are found to be descended five millimeters or more beneath the level of foramen magnum into the CCJ. In contrast to an oversized cerebellum, research has shown that Chiari malformations are often due to hypoplasia of the posterior fossa resulting in crowding and downward displacement of the cerebellar tonsils [32–36]. Some authors have also attributed cerebellar tonsillar ectopia to faulty craniospinal hydrodynamics [181–185]. While further studies need to be done before drawing conclusions, recent research has shown a possible connection between cerebellar tonsillar ectopia and structural strains due to trauma [205, 206]. Structural strains can also occur due to an abnormal clivoaxial angle (craniocervical angulation) and intervals, including retroflexed odontoid, which are often associated with Chiari malformations [36, 207]. Among other things, structural strains of the CCJ affect the dura mater, which can cause displacement (ectopia) of the brainstem and deformation of the neurovascular and CSF tunnels and pathways. An abnormal clivoaxial angle or retroflexed odontoid can cause cervicomedullary kinking and compression of the ventral epidural space of the neural canal of the CCJ. In any case, regardless of the cause, Chiari malformations and coning of the brainstem cause blockage of CSF flow through the CCJ. They also compress the epidural space which contains the vertebral veins that connect to the emissary veins and dural sinuses used to drain the brain during upright posture. The most abundant vertebral veins are found in the anterior aspect of the canal.

Although still hotly debated, most of the CSF in spinal cord and cisterns eventually flows up through the subarachnoid space and CCJ toward the top of the brain where it is absorbed back into venous circulation by the arachnoid granulations. The arachnoid granulations are special one-way valves that develop during infancy as a child starts to hold its head up, crawl, and walk. While they can be found in other dural sinuses as well, the bulk of arachnoid granulations are found at the top of the brain in humans and drain into the venous lacunae and superior sagittal sinus [198]. In quadrupeds the arachnoid granulations are predominantly found in the transverse sinuses. Their locations are most likely driven by pressure gradients associated with head posture and locomotion. The force of flow through the arachnoid granulations is strong enough to leave deep impressions on the roof of the cranial vault. Researchers have also argued and shown that a significant amount of CSF flows into the capillary veins to exit the subarachnoid space. In either case, the absorption of CSF by the arachnoid granulations or by the capillary veins is affected by downstream venous pressure and flow. An increase in venous pressure in the superior sagittal sinus decreases the CSF pressure gradient, which decreases flow and increases CSF volume in the brain. Conversely, venous drainage is affected by the free flow of CSF. An increase in CSF volume in the brain can compress veins and decrease drainage [72–79, 190–204]. Malformations, misalignments, and deformation of the CCJ compress the vertebral veins, which may affect CSF flow.

In addition to flow within the craniospinal compartment, research has further shown that CSF flows along cranial nerve roots, especially the olfactory and optic nerves. In fact, enlargement of the optic nerve sheath is a sign of increased CSF volume and possible hydrocephalus. CSF can also be seen in the trigeminal and other cranial nerves as well. Alternatively CSF can exit the cranial vault and flow through the CCJ into the subarachnoid space of the cord. As in the brain, there is ongoing debate about optional routes for CSF to leave the subarachnoid space of the cord including pathways that follow the nerve roots of the cord similar to cranial nerves in the brain. The bulk of CSF in the subarachnoid space of the cord, however, appears to return to the cranial vault and exit through the arachnoid granulations [190–204]. The CCJ is thus a potential choke point for CSF flow between the cranial vault and spinal canal.

## 4. Hydrocephalus, Hydromyelia, and CCJ

Classic hydrocephalus is an increase in CSF volume coupled with ventriculomegaly. In children, hydrocephalus is typically, but not always, associated with an increase in intracranial pressure (ICP) and head size. For the most part, hydrocephalus is considered a childhood condition. Normal pressure hydrocephalus (NPH) is seen mostly in adults but it is sometimes seen in children as well. NPH is associated with increased CSF volume, ventriculomegaly, and normal or slightly elevated ICP. The size of the head is unaffected in adults. The decrease in compliance of the skull most likely puts a damper on ICP in adults with NPH compared to the highly compliant skulls seen in infants and young children. Despite the development of shunts, decades of research, and recent advances in brain imaging we know very little about the role of faulty craniospinal hydrodynamics in hydrocephalus.

According to Rekate a new definition for hydrocephalus is long overdue and so he proposed one as a starting point for discussion in which he maintains that “*hydrocephalus is an active distension of the ventricular system of the brain resulting from inadequate passage of CSF from its point of production within the cerebral ventricles to its point of absorption into the systemic circulation*” [208, 209]. Because it requires an active process of production and an inadequate passage of CSF flow, Rekate's definition omits hydrocephalus due to other causes, such as vasogenic and cytotoxic edema, as well as brain atrophy, which causes normal pressure hydrocephalus ex vacuo in which CSF volume increases to fill the void. Since it further includes ventriculomegaly it also omits idiopathic intracranial hypertension without ventriculomegaly. Moreover, it omits conditions associated with increased extraventricular CSF volume and enlargement of the subarachnoid spaces and cisterns without ventriculomegaly, such as those seen in certain cases of Dandy-Walker complex and Parkinson's plus syndromes called multisystem atrophy and olivopontocerebellar atrophy.

Other definitions for hydrocephalus have also been proposed over the years. One definition previously proposed by Raimondi is that hydrocephalus is a pathologic increase in CSF volume independent of hydrostatic or barometric pressure that can be classified as either intra- or extraparenchymal. Intraparenchymal hydrocephalus is essentially the same as cerebral edema, which is an increase in interstitial fluids in the parenchyma. Extraparenchymal hydrocephalus can be further broken down into intraventricular, cisternal, and subarachnoid space forms [210].

Some authors maintain that hydrocephalus is simply cerebral edema regardless of the cause or location of the excess CSF volume [208–211]. This is probably the broadest definition as it includes vasogenic and cytotoxic edema, which have nothing to do with blockage of CSF flow. Interestingly the term hyperemic hydrocephalus was recently introduced to describe a condition in which venous outflows fail to match intracranial arterial inflows [78]. Moreover, studies suggest that hyperemic hydrocephalus seen in children and idiopathic intracranial hypertension seen in adults may both be due to venous insufficiency. Idiopathic intracranial hypertension has further been associated with a decrease in the capacity and compliance of the vertebral veins in the spinal canal. In this regard, females tend to have smaller spinal canals and less venous capacity compared to males. The internal volume of the female pelvis is also smaller and further crowded by ovaries and a uterus that swell during menstruation. Obesity and pregnancy increase pressure on the abdominal veins. Increased pressure against the abdominal and vertebral veins may affect the vertebral venous plexus similar to a Valsalva maneuver and cause an increase in ICP. Thus females may be predisposed to idiopathic intracranial hypertension due to their physiology along with the smaller design of the spine and pelvis.

The generally accepted theory is that, aside from frank blockage, such as stenosis of the cerebral aqueduct, the cause of hydrocephalus is due to insufficient absorption, which occurs primarily by way of the arachnoid granulations, as well as arterial and venous capillary routes. According to Greitz, however, the bulk CSF flow theory fails to explain key issues regarding communicating hydrocephalus, such as what causes the ventricles to enlarge, why CSF pressure remains normal in certain cases, and why some patients improve with shunts or endoscopic third ventriculostomy [212]. Greitz thus proposed his hydrodynamic theory in which the patent cerebral aqueduct in communicating hydrocephalus is simply too narrow (insufficient) to adequately drain the lateral and third ventricles. Subsequent saturation of the brain due to hydrocephalus and edema causes a decrease in compliance. The loss of compliance of the veins in the subarachnoid space decreases their buffering capacity. Consequently, high pulsatile arterial pressure waves are transmitted from the subarachnoid space to delicate tissues of the brain parenchyma, as well as the walls of the ventricles in the core of the brain and brainstem. The turgor of the ventricles resists deformation from the incoming forces. The periventricular parenchymal tissues and blood vessels caught between the expanding brain and ventricles take the brunt of the force from the increased arterial pulsations [80–84, 213, 214]. Chronic increased pulsatility can cause damage, atrophy, and subsequent ventriculomegaly. Greitz maintains that the larger pressure waves in the parenchyma cause an overall distention of the brain and subsequent compression of the cortex and periventricular areas by the skull and ventricles, respectively. In contrast to the skull, the ventricles can expand. Chronic increased pressure waves causing tension, compression, and shear stresses of periventricular tissues may lead to atrophy and subsequent enlargement of the ventricles. This would explain why periventricular structures are often affected in hydrocephalus and neurodegenerative diseases and associated with periventricular hyperintensity signals on MRI. The outcome of chronic compression of the brain is enlargement of the ventricles and narrowing of the subarachnoid space. Greitz maintains that endoscopic third ventriculostomy decreases the destructive impact of increased systolic pressure waves in the brain parenchyma by bypassing the restriction and venting CSF through the surgical stoma in the floor of the third ventricle. Fenestration increases the drainage capacity of the third ventricle and dissipates the intraventricular CSF volume, velocity, and pulsatility, which decreases the water hammer effect [212]. Conversely, Rekate proposed that ventriculomegaly may be due to loss of brain turgor and softening of the brain associated with aging [187, 208, 209].

Hydrocephalus in children is typically attributed to blockage of CSF flow somewhere between the ventricles where it is produced and the arachnoid granulations where it is absorbed into venous circulation, which meets the criteria proposed by Rekate's definition. In contrast to children with hydrocephalus, however, blockage of CSF flow is rarely found in adults with normal pressure hydrocephalus (NPH), which is also known as the Hakim-Adams syndrome. Consequently, researchers have long suspected that NPH might be due to insufficient venous drainage of the brain by the dural sinuses [77, 78, 215–224]. Blockages of dural sinuses, however, are likewise rarely found. Earlier research, however, was limited to the dural sinuses and essentially stopped at the cranial vault.

In 1988 this author proposed that neurodegenerative diseases may be due to obstruction and back pressure in the accessory drainage system of the brain as it passes through the CCJ to connect to the vertebral veins [20, 21]. Obstruction can occur due to many types of malformations and misalignments of the CCJ. More recently, in 2009, Zamboni et al. proposed that MS can be caused by venous insufficiency due to stenosis or faulty valves in jugular veins [222]. The many different types of malformations and misalignments of the CCJ that can potentially cause vertebral venous insufficiency, however, far outnumber those due to jugular insufficiency [31–53, 85–87]. Moreover, the vertebral veins are the primary drainage routes used to drain the brain during upright posture [20–30].

One of the primary causes of hydrocephalus due to blockage of CSF flow is stenosis or mass effects causing compression of the cerebral aqueduct that links the third and fourth ventricle. Current research, however, suggests that certain cases of communicating hydrocephalus in children may be due to insufficient venous drainage of the brain. Among other things, researchers now suspect that insufficient venous drainage can cause stenosis of the cerebral aqueduct due to increased CSF volume and pressure in the parenchymal spaces surrounding the midbrain resulting in compression of the aqueduct [223, 224]. In this regard, the cerebral aqueduct passes through the midbrain, which is surrounded by the suprasellar and superior cerebellar (quadrigeminal) cisterns. An increase in CSF volume in the suprasellar and superior cerebellar cisterns (extraparenchymal cisternal hydrocephalus) may play a role in certain cases of aqueductal stenosis and hydrocephalus (ventriculomegaly).

Further adding to the theory regarding the role of the CCJ in faulty craniospinal hydrodynamics and neurodegenerative diseases, in 2008 Williams proposed a unifying hypothesis for the cause of hydrocephalus, Chiari malformations, syringomyelia, anencephaly, and spina bifida based on dissociation of CSF flow between the cranial vault and spinal canal [225, 226]. Typically, the two compartments are connected and act in concert which allows for an ebb and flow of CSF between the cranial vault and spinal canal based on pressure gradients. Under normal conditions, excess blood and CSF volume in the cranial vault are diverted into the epidural vertebral veins of the spinal canal and the subarachnoid space of the cord. Conversely, the proper volume and pressure of CSF in the subarachnoid space of the cord is necessary to maintain the correct volume of CSF in the cisterns of the cranial vault in order to provide buoyancy of the brainstem and prevent coning into the foramen magnum. According to Williams' hypothesis, Chiari malformations cause blockage of CSF flow through the CCJ resulting in dissociation of CSF flow in the cranial and spinal compartments. The dissociation of the compartments causes them to behave independently [225, 226] resulting in increased pulsatility and CSF pressure waves that can be potentially destructive. According to Williams' theory, blockage of CSF flow between the cranial vault and spinal canal may play a role in hydrocephalus and edema.

Among other things, Williams also suggests that relatively lower spinal canal pressure in females in utero allows the cranial vault to close sooner potentially resulting in hypoplasia of the posterior fossa and disposition to crowding of the brainstem and cerebellum resulting in a Chiari malformation [43, 226]. The Chiari malformation causes dissociation of CSF flow and subsequent hydrocephalus. Conversely, greater spinal canal pressure and larger cisterns in males keep the cranial vault pressurized and expanding in utero allowing for a larger cranial vault. Greater spinal canal pressure and larger cisterns, however, may make males susceptible to higher and more destructive pressure waves in the cisterns, which may play a role in autism [43, 226, 227]. Interestingly, research by Shahinian and others regarding obstetrical factors governing the etiopathogenesis of lambdoid synostosis suggests that increased infant head size coupled with early labor along with engagement and contact with a smaller maternal pelvis may play a role in craniosynostosis seen more frequently in males [228]. Craniosynostosis is associated with hypoplasia of the posterior fossa, jugular foramen, and foramen magnum, as well as Chiari malformations that can block blood and CSF flow and cause hydrocephalus. Lastly, according to this author's theory, loss of intracranial compliance due to obstruction of venous blood and CSF outflow in the CCJ may similarly cause increased pulsatility and pressure waves in the cisterns. It is possible that increased pulsatility and pressure waves in the cisterns may play a role in neurodegenerative disorders associated with enlargement of the cisterns and atrophy of the brainstem seen in adults with Parkinson's plus conditions including olivopontocerebellar atrophy, multisystem atrophy, and progressive supranuclear palsy, as well as certain cases of Dandy-Walker Complex, such as mega cisterna magna seen in children.

Some authors have shown a causal relation between low brain blood flow and symptoms of NPH. They surmise that clinical deterioration is probably due to impaired periventricular blood flow associated with interstitial edema, ependymal disruption, microvascular infarctions, gliosis, and neuronal degeneration. Neuronal injury may result from several factors such as (1) mechanical stretching of the anterior cerebral arteries over the corpus callosum due to ventriculomegaly; (2) periventricular impairment of the blood brain barrier; and (3) reduced CSF turnover with decreased elimination of neurotoxic substances, such as *β*-amyloid, tau-protein, and proinflammatory cytokines. Reduction in blood flow in the anterior cerebral arteries decreases blood flow to the frontal regions resulting in local ischemia and possibly clinical signs. Decreased blood flow could also lead to periventricular axonal dysfunction. Prolonged periventricular ischemia would eventually result in myelin degeneration and irreversible axonal loss, possibly explaining why some patients with NPH do not improve after a shunt. The decrease in CSF clearance may also explain the high cooccurrence of Alzheimer-like changes in the cortex of patients with idiopathic NPH. A possible association with Alzheimer's disease, particularly when there is concurrent arterial hypertension and cerebral arteriosclerosis, may explain why many NPH cases remain with severe cognitive and motor deficits after shunting, even when ventricular size decreases postoperatively [68, 71, 73].

Among other things, hydrocephalus compresses the cortical bridging veins against the cranial vault, which can further increase venous insufficiency. Compression of the veins and decreased venous outflow also decreases cerebral perfusion pressure and blood flow in the brain [71, 186, 229]. In addition to veins, hydrocephalus compresses smaller arteries in the brain parenchyma, such as those surrounding the ventricles. Decreases in blood flow due to a decrease in perfusion pressure or compression of arteries can result in ischemia. Ischemia is a major suspect in the cause of atrophy and subsequent normal pressure hydrocephalus ex vacuo in adults by creating a void for CSF to fill [229–232]. Ventriculomegaly also causes potentially destructive tension, compression, and shear stress of susceptible periventricular tissues. Lastly, according to Foltz [80, 81], Greitz [14, 82, 212], Madsen [16, 84], Bateman [231, 233], and others, hydrocephalus causes increased CSF pulsatility and high pressure waves resulting in destructive water hammer effects on delicate parenchymal tissues. In this author's opinion, the enlarged cisterns (extraparenchymal cisternal hydrocephalus), such as those seen in Dandy-Walker complex and Parkinson's plus syndromes, may likewise be due to destructive pressure waves [20, 21, 28, 91, 98, 102, 128, 234].

In addition to ischemia, edema, structural strains, and pressure waves, hydrocephalus causes sluggish CSF flow. As arteries and veins leave the subarachnoid space the pia mater peels away and follows their course into the parenchyma. This creates a perivascular space (Virchow-Robins space) between the pia mater and tunica adventitia of the blood vessels. CSF flows out of the subarachnoid spaces through the perivascular spaces and into the brain parenchyma where it mixes with interstitial fluids. Interstitial fluids flow out of the parenchyma along perivascular pathways. Since the brain has no lymphatic vessels or glands and it is not connected to the lymphatic system, it instead uses the perivascular spaces to move nutrients in and wastes out. Because of this, the author has also long maintained that the perivascular spaces serve as the lymphatic system of the brain and that sluggish CSF flow may play a role in pathology of neurodegenerative diseases and the increase in the presence of breakdown byproducts, viruses, bacteria, and heavy metals such as aluminum and iron [20, 21, 28, 91, 98, 102, 128, 234]. More recently it was discovered that in addition to providing pathways for the removal of wastes, the glial support cells that form the perivascular spaces also act as macrophages, which led to the term glymphatic system. Researchers now suspect that protein aggregation diseases, such as Alzheimer's and Parkinson's and the accumulation of beta amyloids and alpha synuclein, respectively, may be due to sluggish CSF flow [235–239]. The aggregation of wastes and breakdown byproducts, as well as bacteria and viruses due to sluggish CSF flow, may play a role neurodegenerative processes resulting in atrophy and subsequent ventriculomegaly.

In this regard, the ventricles are the high side of the CSF pressure gradient. The low side of the CSF pressure gradient is venous pressure in the superior sagittal sinus. Superior sagittal sinus venous pressure and flow is affected by posture, respiration, and movement. It is also affected by downstream pressure in the internal jugular and vertebral veins. Obstruction of flow in the internal jugular veins, such as in Queckenstedt's test, is transmitted to the dural sinuses and increases ICP. Likewise, back pressure against abdominal and thoracic veins, such as in a Valsalva maneuver, is transmitted via the vertebral veins to the dural sinuses, which increases ICP. During upright posture the brain preferentially drains into the vertebral venous plexus (VVP). Blockage of the VVP in the CCJ due to malformations and misalignments may cause chronic venous hypertension, decreased CSF flow, and an increase in CSF volume and pulsatility in the cranial vault.

A syrinx, also known as syringomyelia, is enlargement of a portion of the central canal of the cord. It is the homolog of hydrocephalus and sometimes referred to as hydromyelia. The central canal of the cord is typically connected to the obex of the fourth ventricle. In many cases it is not. There are currently three basic theories regarding the cause of syrinx formation and its maintenance. The first theory, called Gardner's theory [240, 241], is that syrinxes are caused by a water hammer effect due to insufficient drainage through the foramen of Magendie and Luschka. The second cause is based on Oldfield's theory, which maintains that the descent of the cerebellum into the foramen magnum causes a piston-like effect that forces fluids into the syrinx [242, 243]. The third theory is William's theory. According to William's theory, syringomyelia is caused by dissociation of craniospinal hydrodynamics [225, 244]. A similar theory was later proposed by Williams, mentioned previously, regarding Chiari malformations and syringomyelia. According to Williams, dissociation of craniospinal hydrodynamics causes lower pressure in the subarachnoid space relative to the central canal [226]. The decrease in flow through the foramen magnum to the spinal canal decreases the volume and pressure of CSF in the surrounding subarachnoid space of the cord that would normally oppose enlargement of the syrinx. It also decreases venous blood flow and volume in the epidural vertebral veins. Both William and Williams further propose that an insufficient return of CSF flow from the spinal canal to the cranial vault can cause CSF volume, pressure, and pulsatility to increase the subarachnoid space of the cord and lumbar cistern, which can lead to spina bifida and meningomyeloceles [225, 226, 244].

Most syrinxes are found in the cervical and thoracic cord. They are frequently associated with Chiari malformations and attributed to blockage and faulty CSF flow through the CCJ [242, 245, 246]. A syrinx located in the lower one-third of the cord is called a terminal syrinx. A terminal syrinx is often associated with a tethered cord and likewise attributed to faulty CSF flow. Tethered cords are also associated with Chiari malformations and scoliosis [247, 248]. A holocord is a syrinx that extends the full length of the cord from the cervicomedullary junction to the terminal ventricle located at the caudal end of the central canal in the conus medullaris of the thoracolumbar spine. In addition to tumors, holocords are likewise frequently associated with Chiari malformations [244, 249–251]. Complex Chiari is a term recently coined by Brockmeyer. A complex Chiari is a condition in which CTE is further complicated by malformations and abnormalities of the CCJ such as abnormal clivoaxial angle, retroflexed odontoid, occipitalization of atlas, basilar invagination, cervicomedullary kinking, syringomyelia, and scoliosis [207].

Although rare, syrinx and holocord have more recently been associated with previously unsuspected conditions such as (1) a dural arteriovenous fistula at the level of the foramen magnum [252], (2) cervical spondylosis [253], (3) stenosis of the thoracic spine [254], (4) a sacral arachnoid cyst [255], and (5) a tethered cord [256]. All of these cases were likewise attributed to altered CSF flow and pressure gradients in the spinal canal and cord. Surprisingly, surgical correction of the above conditions often resulted in complete resolution of the syrinx. Perhaps the most perplexing case was the sacral arachnoid cyst since most syrinxes are classically attributed to blockage of CSF flow in the CCJ. All of these cases underscore the complexity of hydrodynamics in the spinal canal, which is similar to those seen in the cranial vault but beyond the scope of this paper.

Interestingly, in 2003 Chang and Nakagawa proposed a hypothesis regarding the pathogenesis of syringomyelia in which the cisterna magna functions as a shock absorber against the pulsatile CSF pressure waves emanating from within the brain [257]. The loss of buffering capacity of the cisterna magna shifts pressure to the central canal. The increase in pulsatility in the central canal leads to syrinx formation in patients with Chiari I malformation. As mentioned above, according to these authors' hypothesis, an increase in CSF volume in the cisterns may cause potentially destructive increases in pulsatility and pressure waves surrounding the brainstem, which may cause or contribute to atrophy of the brainstem seen in Dandy-Walker complex and Parkinson's plus syndromes mentioned previously. In any case, the CCJ is the critical link between hydraulics in the cranial vault and spinal canal that can affect CSF flow and pulsatility in the brain and cord.

## 5. Discussion

For the most part, the cranial vault is a closed container with little room for expansion or compliance. The spinal canal typically has much greater compliance due to the large lumbar cistern and absence of the cord. CSF volume and pressure in the cranial vault are directly related and pressure increases exponentially with increases in volume. The total volume of the cranial vault is a combination of brain, arterial blood, venous blood, CSF, and ISF. Increases in the volume of blood, CSF, brain, or space occupying lesions, such as tumors, cause an increase in ICP. Arterial volume and pressure in the cranial vault fluctuate causing pulsatile flow and pressure waves in the brain. Arterial flow can be increased or decreased by cerebral autoregulatory circulatory controls located in the cavernous and suboccipital cavernous sinus in order to maintain a relatively stable arterial supply. Stable blood flow is further maintained by proportionate outflows of venous blood and CSF from the cranial vault. A decrease in venous outflow reduces the volume of arterial blood that can enter the brain. It also increases turgor and decreases compliance, which decreases capacity of the veins in the subarachnoid space to buffer incoming arterial pressure fluctuations before they are transmitted to the parenchyma.

An increase in brain, blood, or CSF volume in the cranial vault, which occurs in space occupying lesions, hemorrhages, and hydrocephalus, can cause compression of the bridging veins and venous hypertension. Venous hypertension decreases cerebral perfusion pressure and thus arterial inflows. Chronic decreases in arterial flow can lead to oxidative stress, ischemia, and subsequent atrophy resulting in a compensatory increase in CSF volume, which occurs in normal pressure hydrocephalus ex vacuo. Atrophy can also be caused by compression, tension, and shear stresses acting on the brain and blood vessels due to hydrocephalus and increased pulsatility. In either case, enlarged ventricles and cisterns are a sign of hydrocephalus and an increase in CSF volume that can be caused by atrophy or chronic strains and deformation of the brain and brainstem.

In brief, craniospinal hydrodynamics are a complex interaction between brain, blood, and CSF in the relatively closed compartments of the cranial vault and spinal canal compounded by cardiac cycles and arterial pulsations that cause continuous fluctuations in blood volume and ICP. Faulty craniospinal hydrodynamics have been associated with hydrocephalus, anomalies of the CCJ, Chiari malformations (cerebellar tonsillar ectopia), craniosynostosis, craniofacial anomalies, and Dandy-Walker complex in children. Faulty craniospinal hydrodynamics may also play a role in neurodegenerative diseases such as Alzheimer's, Parkinson's, multiple sclerosis, dementia, and motor neuron diseases, as well as other neurological conditions including migraines, silent-strokes, seizures, psychosis, schizophrenia, depression, and mania. Manual and surgical methods for correcting obstructions, as well as manipulation of blood and CSF flow, may help to restore or improve faulty craniospinal hydrodynamics in certain cases and decrease the prevalence, progression, and severity of neurodegenerative and other neurological conditions. Further studies using upright and cine MRI coupled with computer modeling are needed to determine the role of malformations and misalignments of the CCJ and spondylosis, stenosis and scoliosis in the lower spine in faulty craniospinal hydrodynamics, and neurodegenerative and neurological conditions, as well as the impact of the manual, mechanical, and surgical correction of structural strains and faulty craniospinal hydrodynamics on patient pathology and symptomatology.
